# A Power Allocation Scheme for MIMO-NOMA and D2D Vehicular Edge Computing Based on Decentralized DRL

**DOI:** 10.3390/s23073449

**Published:** 2023-03-25

**Authors:** Dunxing Long, Qiong Wu, Qiang Fan, Pingyi Fan, Zhengquan Li, Jing Fan

**Affiliations:** 1 School of Internet of Things Engineering, Jiangnan University, Wuxi 214122, China; 2 State Key Laboratory of Integrated Services Networks, Xidian University, Xi’an 710071, China; 3 Qualcomm, San Jose, CA 95110, USA; 4 Department of Electronic Engineering, Beijing National Research Center for Information Science and Technology, Tsinghua University, Beijing 100084, China; 5 Changzhou Key Laboratory of 5G + Industrial Internet Fusion Application, Changzhou 213001, China; 6 University Key Laboratory of Information and Communication on Security Backup and Recovery in Yunnan Province, Yunnan Minzu University, Kunming 650500, China

**Keywords:** vehicular edge computing (VEC), power allocation, MIMO-NOMA, D2D, deep deterministic policy gradient (DDPG), decentralized

## Abstract

In vehicular edge computing (VEC), some tasks can be processed either locally or on the mobile edge computing (MEC) server at a base station (BS) or a nearby vehicle. In fact, tasks are offloaded or not, based on the status of vehicle-to-infrastructure (V2I) and vehicle-to-vehicle (V2V) communication. In this paper, device-to-device (D2D)-based V2V communication and multiple-input multiple-output and nonorthogonal multiple access (MIMO-NOMA)-based V2I communication are considered. In actual communication scenarios, the channel conditions for MIMO-NOMA-based V2I communication are uncertain, and the task arrival is random, leading to a highly complex environment for VEC systems. To solve this problem, we propose a power allocation scheme based on decentralized deep reinforcement learning (DRL). Since the action space is continuous, we employ the deep deterministic policy gradient (DDPG) algorithm to obtain the optimal policy. Extensive experiments demonstrate that our proposed approach with DRL and DDPG outperforms existing greedy strategies in terms of power consumption and reward.

## 1. Introduction

With the development of the Internet of vehicles (IoV), autonomous vehicles are becoming increasingly popular. At the same time, a series of smart vehicular user (SVU) devices and applications installed on autonomous vehicles have emerged. Communication among these SVU devices and applications is extremely popular [1,2,3,4,5]. Therefore, to reduce the burden of SVUs, vehicular edge computing (VEC) has been introduced to process tasks offloaded by SVUs, while ensuring low processing latency for these tasks [6,7,8]. When SVUs have tasks to process, they can choose to process these tasks locally or offload them to a mobile edge computing (MEC) server on a VEC vehicle or at a base station (BS) [9,10]. Such task offloading incurs multiple kinds of power consumption. To be clear, in this paper, the power consumed for offloading to the BS is defined as vehicle-to-infrastructure (V2I) processing power, and the power consumed for offloading to a VEC vehicle is defined as vehicle-to-vehicle (V2V) processing power. In addition, SVUs also process tasks on their local CPUs; the task processing power consumed by the local CPU of an SVU is defined as local processing power in this paper.

In this paper, V2I communication is assumed to be based on the multiple-input multiple-output and nonorthogonal multiple access (MIMO-NOMA) technology, due to high spectrum utilization and channel capacity. However, in the MIMO-NOMA system, the interference of SVUs with each other and the mobility of SVUs lead to uncertain channel conditions [11].

V2V communication is assumed to be based on device-to-device (D2D) technology, which is a core technology for smart cities, high-quality video streaming, and disaster-relief networks. It offers various advantages in terms of fairness, energy efficiency, and spectral efficiency [12,13,14,15]. In the D2D communication scenario considered in this paper, each SVU communicates only with a specific VEC vehicle, and the communication distance remains constant. Therefore, V2V communication is working on the interference-free channel conditions [16].

In a real scenario, task arrival will be random. In this case, the latency and power consumption of the SVUs for processing the tasks will also be uncertain [11]. For example, when the V2I channel conditions are relatively worse, SVUs should choose to process their tasks either locally or through V2V communication to reduce latency and power consumption. Considering the randomness of task arrival and the uncertainty of the V2I channel conditions, it is necessary to design an optimal power-allocation scheme with the aim of minimizing latency and power consumption.

This paper employs the deep reinforcement learning (DRL) framework to design such a scheme. Since the deep deterministic policy gradient (DDPG) algorithm is suitable for solving problems in the continuous action space, we will employ this characteristic of the algorithm to solve the problem. In most previous work, the BS was responsible for collecting global information, including the SVU state, and then determining the action of each SVU; however, the centralized approach incurs higher power consumption and larger latency [17,18,19,20,21,22,23,24,25,26,27,28,29,30]. Only a few works have adopted decentralized DRL frameworks to design related schemes in which each SVU observes its own surrounding environment to determine its action. In this way, it can effectively reduce the overall latency and power consumption [31,32]. However, to the best of our knowledge, the coexistence of MIMO-NOMA-based V2I communication and D2D-based V2V communication was not considered in the VEC system based on the decentralized DRL.

In this paper, we propose a power-allocation model in VEC based on decentralized DRL to improve power consumption and latency, considering the coexistence of MIMO-NOMA-based V2I communication and D2D-based V2V communication, as well as the randomness of task arrival, the channels interference in MIMO-NOMA and the mobility of SVUs. (The source code has been released on https://github.com/qiongwu86/MIMO-D2D (accessed on 19 February 2023). The main contributions of this article are summarized as follows.

(1) We propose a power allocation model in VEC based on the decentralized DRL, defining the action function, state function, and reward function. The DDPG algorithm is employed to deal with the continuous action space problem and to guide the model to learn the optimal policy.
(2) Performance testing of the trained model in a large number of experiments shows that the proposed approach outperforms other existing ones.

The rest of this paper is organized as follows. Section 2 reviews some related work. Section 3 describes the system model. Section 4 presents the design of the DRL framework and the related functions. Section 5 describes the DDPG algorithm used for model training. We present some simulation results to demonstrate that our proposed approach outperforms other existing ones in Section 6. Finally, Section 7 concludes the paper.

## 2. Related Work

In this section, we review some work on D2D and MIMO-NOMA technology in MEC and VEC based on DRL.

### 2.1. D2D and MIMO-NOMA Technology in MEC and VEC

Many works have considered the application of MIMO-NOMA and D2D technology in MEC and VEC systems. In [33], Ding et al. proposed a multiuser MIMO (MU-MIMO) MEC system with the goal of optimizing the system cost, power consumption and latency. In [11], Zhu et al. constructed a VEC system based on MIMO-NOMA technology, in which vehicles can choose to process tasks either locally or offload them to the BS via the MIMO-NOMA channels. In this system, the DDPG algorithm was employed to optimize latency and power consumption. In [34], Liu et al. designed a millimeter-wave D2D MEC model as the basis of an optimal algorithm for task assignment. In [35], Li et al. proposed a MEC system supporting D2D. It formulated a two-stage optimization algorithm with the goal of improving resource utilization and network capacity. However, these works did not consider the coexistence of MIMO-NOMA and D2D technology in MEC and VEC.

### 2.2. V2V and V2I Communication in VEC

Some research works have also employed V2V and V2I communication in VEC systems. In [11], Zhu et al. considered V2I communication based on MIMO-NOMA in a VEC system. In [1], Raza et al. proposed a 5G-based VEC system in which the V2I communication was based on the millimeter-wave mode. In [36], Zhang et al. constructed a software-defined networking (SDN)-assisted VEC system with the goal of optimizing the system overhead while considering 802.11p-based V2I communication and V2V communication. In [37], Bai et al. designed a VEC system containing vehicular cloudlets and considered D2D-based V2V communication. However, these works did not consider the coexistence of MIMO-NOMA-based V2I communication and D2D-based V2V communication in VEC systems.

### 2.3. DRL-Based Resource Allocation in VEC

There have been many studies on DRL-based resource allocation in VEC. In [38], Ning et al. proposed a VEC system consisting of multiple roadside units (RSUs), SVUs and a single BS. With the aims of optimizing the system cost, the DDPG algorithm was employed to obtain the optimal resource allocation scheme. In [39], Ren et al. proposed a VEC system consisting of multiple RSUs, BSs, and SVUs. A centralized resource allocation mechanism based on DRL was designed to obtain an optimal network resource-allocation scheme. In [23], Liu et al. designed a semi-Markov process-based scheme for maximizing VEC network utility and employed the deep Q-learning network (DQN) algorithm to achieve optimal resource allocation. However, none of the above works considered decentralized DRL-based VEC systems.

Some works have also considered decentralized DRL-based VEC systems. In [31], Ye et al. constructed a VEC system with both V2I communication and V2V communication, in which SVUs used the DQN algorithm to select the transmission band for their tasks, thus optimizing the system capacity and latency. In [11], Zhu et al. designed a VEC system consisting of a BS and SVUs, considering the mobility of SVUs and MIMO-NOMA technology. The SVUs employed the DDPG algorithm to allocate processing power, thus optimizing the latency and power consumption. However, [11] did not consider the presence of V2V communication, while [31] did not consider the mobility of SVUs, MIMO-NOMA, and D2D technology.

As seen from the above review, no previous works have considered the coexistence of MIMO-NOMA-based V2I communication and D2D-based V2V communication in the power allocation problem for decentralized DRL-based VEC systems. This motivates us to start this work. We shall show it in detail.

## 3. System Model

**Figure 1:**
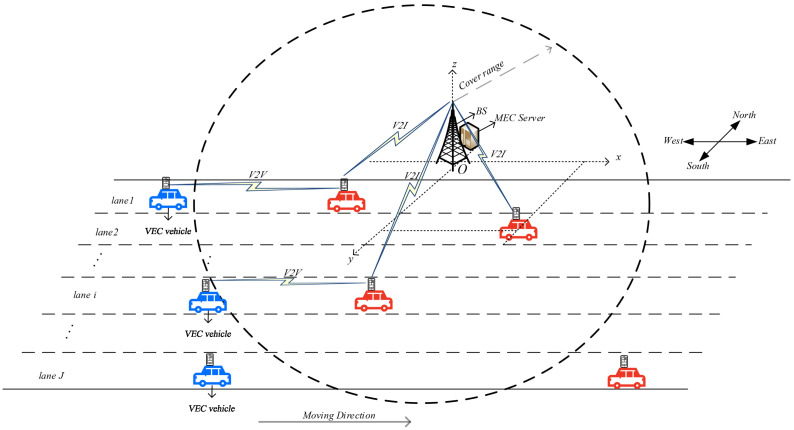
System model.

The system model is shown in Figure 1. A MEC server is placed on each VEC vehicle and at the BS with multiple antennae. Based on the axial distance to the BS, the VEC server coverage is divided into *J* lanes, where SVUs may move at different speeds in different lanes. The time duration for which SVUs in lane *j* remain within the communication range of the BS is divided into $N_{j,m}$ slots, and the length of each slot is $\tau_{0}$. In each slot, tasks arrive randomly in the buffers of the SVUs. At the same time, each SVU allocates local processing power, V2I processing power, and V2V processing power to process tasks either locally or offload them to the MEC server. In addition, the V2I channel conditions continuously change due to the mobility of SVUs and the channel’s interference in MIMO-NOMA. In contrast, V2V communication is based on D2D technology, and it is assumed that each SVU communicates only with a specific VEC vehicle in the same lane, and each VEC vehicle processes tasks for only one specific SVU; thus, the V2V channel conditions can be treated as time invariant. In V2I communication, each SVU first transmits tasks to the BS; the BS processes the tasks and employs the zero-forcing (ZF) technique to detect the signal and noise associated with each SVU from all SVU signals and then obtains the signal-to-interference-plus-noise ratio (SINR) of each SVU for V2I communication. In the next slot, the BS transmits the SINR of each SVU to that SVU. In contrast to the traditional scheme of centralized DRL, in this work, each SVU can determine its own power allocation according to its own observations of the environment. It is a decentralized mode. In the following, we shall introduce the system’s mobility model, task-computation model, and communication model and describe the relevant environmental information, such as the buffer capacity of SVU *k*, the SINRs of V2I communication and V2V communication, and the position of SVU *k*. The notations used in this article can be seen in Table 1.

### 3.1. Mobility Model

Let $P_{k,j}\left(n\right)$ denote the position of SVU *k* in lane *j* in slot *n*. We establish a three-dimensional right-angle coordinate system as shown in Figure 1, where the BS is the origin point, the *x*-axis direction is the travel direction of SVU *k*, the *y*-axis represents the vertical distance to the BS, and the *z*-axis represents the height difference to the BS. Let $d_{k,j}\left(n\right)$ and $w_{k,j}$ be the distances between SVU *k* in slot *n* and the BS along the *x*-axis and *y*-axis, respectively. Therefore, $P_{k,j}\left(n\right)$ can be expressed as $\left(d_{k,j}\left(n\right),w_{k,j},0\right)$, where $w_{k,j}$ is equal to the vertical distance of lane *j* from the BS, which can be calculated as

(1) $$w_{k,j}=\left(j-1\right)\cdot w_{d}+w_{1},$$

where $w_{d}$ is the lane width and $w_{1}$ is the distance between the BS and lane 1.

Similar to [40], for simplification, we shall employ the discrete approximation model and assume that the position of SVU *k* is constant within each individual slot because the time duration $\tau_{0}$ of each slot is small. Since the velocity $v_{j}$ of SVU *k* in lane *j* is constant, $d_{k,j}\left(n\right)$ can be expressed as

(2) $$d_{k,j}\left(n\right)=d_{k,j}\left(n-1\right)+v_{j}\tau_{0},$$

where $d_{k,j}\left(n\right)\in \left[-\frac{D}{2},\frac{D}{2}\right]$ and $d_{k,j}\left(1\right)=-\frac{D}{2}$. SVU *k* can determine whether it is within the coverage area of the BS based on its own current position $d_{k,j}\left(n\right)$, which reflects the mobility of SVU *k*.

### 3.2. Communication Model

#### 3.2.1. V2I Communication

The channel matrix between the BS and the SVUs in slot *n* can be expressed as $G\left(n\right)=\left[g_{1,I}\left(n\right),\cdots ,g_{k,I}\left(n\right),\cdots ,g_{M,I}\left(n\right)\right]\in C^{N_{a}\times M}$, where $N_{a}$ is the number of BS antennae and $g_{k,I}\left(n\right)\in C^{N_{a}\times 1}$ is the channel vector between SVU *k* and the BS. In the MIMO-NOMA channels, the signals received by the BS in slot *n* from all SVUs can be expressed as

(3) $$\begin{array}{c} y\left(n\right)=\sum_{k\in M}g_{k,I}\left(n\right)\sqrt{p_{k,I}\left(n\right)}s_{k}\left(n\right)+N_{s}\left(n\right),\\ p_{k,I}\left(n\right)\in \left[0,P_{max,I}\right], \end{array}$$

where $N_{s}\left(n\right)$ is Gaussian white noise and $s_{k}\left(n\right)$ is complex data symbol with unit variance. Here, $p_{k,I}\left(n\right)$ is the V2I processing power of SVU *k* in slot *n*, with $P_{max,I}$ being the maximum V2I processing power of SVU *k*. In addition, $g_{k,I}\left(n\right)$ reflects the path loss of SVU *k* for V2I communication, which can be expressed as [41]

(4) $$g_{k,I}\left(n\right)=g_{k}^{s}\left(n\right)\sqrt{g_{k}^{p}\left(n\right)},$$

where $g_{k}^{s}\left(n\right)$ is the small-scale fading channel gain and $g_{k}^{p}\left(n\right)$ is the large-scale fading coefficient. $g_{k}^{p}\left(n\right)$ reflects the mobility of SVU *k* and is calculated as

(5) $$g_{k}^{p}\left(n\right)=\frac{g_{r}}{{∥P_{k,j}\left(n\right)-P_{B}∥}^{\eta }},$$

where $g_{r}$ is the channel gain at 1 m and η is the path loss exponent. Meanwhile, $P_{k,j}\left(n\right)=\left(d_{k,j}\left(n\right),w_{k,j},0\right)$ is the position of SVU *k* in slot *n*, and $P_{B}=\left(0,0,H_{a}\right)$, where $H_{a}$ is the height of the BS antenna. Note that $P_{k,j}\left(n\right)$ can be calculated from Equations (1) and (2).

The small-scale fading channel gain is initialized as $g_{k}^{s}\left(0\right)∼\mathrm{CN}\left(0,I_{K}\right)$, where $I_{K}$ is an $N_{a}\times N_{a}$ identity vector.

The relationship between $g_{k}^{s}\left(n\right)$ and $g_{k}^{s}\left(n-1\right)$ can be expressed as [42]

(6) $$g_{k}^{s}\left(n\right)=\rho_{k}g_{k}^{s}\left(n-1\right)+\sqrt{1-\rho_{k}^{2}}e\left(n\right),$$

where e(n) is an error vector and we can obtain $\rho_{m}$ as described in [43]. $\rho_{k}$ is the normalized channel correlation coefficient and is correlated with θ, which is the angle between the movement direction of SVU *k*, i.e., the *x*-axis, and the direction of communication, i.e., $P_{B}-P_{k,j}\left(n\right)$. θ is calculated as

(7) $$\theta =\arccos(\frac{x_{0}\cdot \left(P_{B}-P_{k,j}\left(n\right)\right)}{∥P_{B}-P_{k,j}\left(n\right)∥}),$$

where $x_{0}=\left(1,0,0\right)$.

By using Equations (5)–(7), SVU *k* can obtain the channel vector $g_{k,I}\left(n\right)$.

Subsequently, the BS employs the ZF technique [42] to obtain the SINR of SVU *k* for V2I communication, denoted by $\gamma_{k,I}\left(n\right)$, which is calculated as

(8) $$\gamma_{k,I}\left(n\right)=\frac{p_{k,I}\left(n\right)}{{∥g_{k}^{G}\left(n\right)∥}^{2}\sigma_{R}^{2}},$$

where $p_{k,I}\left(n\right)$ is the V2I processing power of SVU *k* in slot *n*, $\sigma_{R}^{2}$ is the noise power, and $g_{k}^{G}\left(n\right)$ is the *k*th row of the pseudoinverse of G(n). Therefore, the relationship between $g_{k}^{G}\left(n\right)$ and $g_{k,I}\left(n\right)$ is

(9) $$g_{k}^{G}\left(n\right)g_{i,I}\left(n\right)=\left\{\begin{array}{c} 1,\;i=k,\\ 0,\;i\neq k. \end{array}\right.$$

Based on Equations (3)–(9), the BS can obtain $\gamma_{k,I}\left(n\right)$ and transmit it to SVU *k* in the next slot. Thus, SVU *k* is able to observe $\gamma_{k,I}\left(n-1\right)$ in the local environment in slot *n*, which reflects the uncertain channel conditions of SVU *k* for V2I communication caused by mobility of SVU *k*.

#### 3.2.2. V2V Communication

Similar to [16], V2V communication is based on D2D technology. Since the channel conditions between SVU *k* and its corresponding VEC vehicle are time invariant, the channel gain between them can be expressed as

(10) $$h_{k,V}=\beta_{0}{f_{i}}^{2}R_{k}^{-\alpha_{h}},$$

where $f_{i}$ is an exponentially distributed random variable with unit mean. $\alpha_{h}$ and $\beta_{0}$ are the path loss exponent and the channel power gain at the reference distance, respectively, for V2V communication. $R_{k}$ is the distance between the communicating vehicles. Since each SVU communicates only with a specific VEC vehicle in the same lane, $R_{k}$ is a constant.

Therefore, the SINR of SVU *k* for V2V communication, denoted by $\gamma_{k,V}\left(n\right)$, is calculated as

(11) $$\gamma_{k,V}\left(n\right)=\frac{p_{k,V}\left(n\right)h_{k,V}}{\sigma_{R}^{2}},$$

where $p_{k,V}\left(n\right)\in \left[0,P_{max,V}\right]$ is the V2V processing power of SVU *k* in slot *n*.

By using Equations (11) and (12), SVU *k* can obtain the SINR for V2V communication in slot *n*, where $\gamma_{k,V}\left(n\right)$ is related only to $p_{k,V}\left(n\right)$, which reflects the fact that the channel conditions of SVU *k* for V2V communication are time invariant.

### 3.3. Task-Computation Model

The relationship between the buffer capacity $B_{k}\left(n\right)$ and $B_{k}\left(n-1\right)$ of SVU *k* in slot *n* is calculated as

(12) $$B_{k}\left(n\right)={\left[B_{k}\left(n-1\right)-\left(d_{k,L}\left(n-1\right)+d_{k,I}\left(n-1\right)+d_{k,V}\left(n-1\right)\right)\right]}^{+}+a_{k}\left(n-1\right),$$

where ${\left[\cdot \right]}^{+}=\max\left(0,\cdot \right)$ and $a_{k}\left(n-1\right)$ is the number of tasks arriving in slot *n*. $d_{k,L}\left(n-1\right)$, $d_{k,I}\left(n-1\right)$, and $d_{k,V}\left(n-1\right)$ are the numbers of tasks processed in slot n−1 for local processing, V2I processing, and V2V processing, respectively. The descriptions of how to calculate $d_{k,L}\left(n-1\right)$, $d_{k,I}\left(n-1\right)$ and $d_{k,V}\left(n-1\right)$ are given below.

#### 3.3.1. Local Processing

Let *L* be the computational intensity of the tasks, i.e., the number of cycles required for the CPU to process one bit. Let $f_{k}\left(n-1\right)$ be the CPU processing frequency of SVU *k* in slot n−1. Therefore, $d_{k,L}\left(n-1\right)$ is calculated as

(13) $$d_{k,L}\left(n-1\right)=\tau_{0}f_{k}\left(n-1\right)/L,$$

where $f_{k}\left(n-1\right)$ is calculated as

(14) $$\begin{array}{c} f_{k}\left(n-1\right)=\sqrt[{3}]{p_{k,L}\left(n-1\right)/\kappa },\\ p_{k,L}\in \left[0,P_{max,L}\right], \end{array}$$

where $p_{k,L}\left(n-1\right)$ is the local processing power of SVU *k* in slot n−1 and κ is a constant that reflects the effective converted capacitance.

#### 3.3.2. V2I and V2V Processing

Since the computational resource of a MEC server is assumed to be sufficient, the latency of a MEC server in processing tasks is negligible. Moreover, the size of the computation result is very small, so the feedback latency is also negligible. Therefore, according to Shannon’s theorem, $d_{k,I}\left(n-1\right)$ and $d_{k,V}\left(n-1\right)$ are calculated as

(15) $$d_{k,I}\left(n-1\right)=\tau_{0}W_{d}\log_{2}\left(1+\gamma_{k,I}\left(n-1\right)\right),$$

(16) $$d_{k,V}\left(n-1\right)=\tau_{0}W_{d}\log_{2}\left(1+\gamma_{k,V}\left(n-1\right)\right),$$

where $W_{d}$ is the bandwidth and $\gamma_{k,I}\left(n-1\right)$ and $\gamma_{k,V}\left(n-1\right)$ are the SINRs of SVU *k* at slot n−1 for V2I communication and V2V communication, respectively.

The buffer capacity $B_{k}\left(n\right)$ of SVU *k* can be calculated from $B_{k}\left(n-1\right)$ based on Equations (12)–(16). Since $B_{k}\left(n\right)$ depends on $a_{k}\left(n-1\right)$, $d_{k,L}\left(n-1\right)$, $d_{k,I}\left(n-1\right)$ and $d_{k,V}\left(n-1\right)$, it reflects the randomness of task arrival and the uncertainty of the channel conditions for V2I communication.

## 4. Problem Formulation

In this section, we describe the DRL-based framework, which consists of state, action, and reward functions. The state is defined based on the environment of each SVU in slot *n*; the action corresponds to the power allocation of each SVU, which is based on a policy μ and the reward is the benefit earned as a result of the action, which is related to the power consumption and latency.

### 4.1. State

In this paper, each SVU observes its surrounding environment to determine its power allocation. The V2I channel conditions for each SVU are uncertain because of the channel’s interference in MIMO-NOMA and the mobility of each SVU. Moreover, the task arrival is random. Based on joint consideration of these two issues, the state is formulated to reflect the uncertainty of V2I channel conditions and the randomness of task arrival.

In the system model, the distance of SVU *k* from the BS along the *x*-axis $d_{k,j}\left(n\right)$ reflects its mobility. In addition, according to Equations (8) and (9), the SINR $\gamma_{k,I}\left(n\right)$ of SVU *k* for V2I communication depends on $g_{k}^{G}\left(n\right)$, which in turn depends on $g_{k,I}\left(n\right)$; thus, we find that $\gamma_{k,I}\left(n\right)$ depends on $g_{k,I}\left(n\right)$. Therefore, $\gamma_{k,I}\left(n\right)$ reflects the uncertainty of the V2I channel conditions. Moreover, according to Equations (12)–(16), the buffer capacity $B_{k}\left(n\right)$ of SVU *k* in slot *n* is a function of $a_{k}\left(n-1\right)$ and $\gamma_{k,I}\left(n-1\right)$, where $a_{k}\left(n-1\right)$ reflects the randomness of the task-arrival rate and $\gamma_{k,I}\left(n-1\right)$ reflects the uncertainty of the V2I channel conditions. Therefore, $B_{k}\left(n\right)$ reflects both the randomness of task arrival and the uncertainty of the V2I channel conditions. Since SVU *k* can observe $d_{k,j}\left(n\right)$, $\gamma_{k,I}\left(n-1\right)$ and $B_{k}\left(n\right)$ in the local environment, the state of SVU *k* in slot *n* can be expressed as

(17) $$s_{k,n}=\left[B_{k}\left(n\right),\gamma_{k,I}\left(n-1\right),d_{k,j}\left(n\right)\right],$$

where $\gamma_{k,I}\left(n-1\right)$ depends on $g_{k,I}\left(n\right)$ and the buffer capacity $B_{k}\left(n\right)$ is related to $\gamma_{k,I}\left(n-1\right)$ and $a_{k}\left(n-1\right)$. Since $g_{k,I}\left(n\right)$ and $a_{k}\left(n-1\right)$ are continuous values, the state space of SVU *k* is continuous.

### 4.2. Action

SVU *k* allocates its local processing power $p_{k,L}\left(n\right)$, V2I processing power $p_{k,L}\left(n\right)$, and V2V processing power $p_{k,L}\left(n\right)$ in accordance with the current state observed in slot *n*. Thus, the action of SVU *k* in slot *n* can be expressed as

(18) $$a_{k,n}=\left[p_{k,L}\left(n\right),p_{k,I}\left(n\right),p_{k,V}\left(n\right)\right].$$

note that similar to [44], we consider the action space of SVU *k* to be continuous.

### 4.3. Reward

The reward is an evaluation based on the previous action. In this paper, we aim to minimize power consumption and latency. As mentioned in Section 3, the latency of task offloading is a constant. Thus, based on Little’s theorem [45], the reward of SVU *k* is defined as

(19) $$r_{k,n}=-\left[\omega_{1}\left(p_{k,L}\left(n\right)+p_{k,I}\left(n\right)+p_{k,V}\left(n\right)\right)+\omega_{2}B_{k}\left(n\right)\right],$$

where $\omega_{1}$ and $\omega_{2}$ are nonnegative weight factors and $\omega_{1}+\omega_{2}=1$.

Accordingly, the cumulative discount reward of SVU *k* can be calculated as

(20) $$J\left(\mu_{k}\right)E_{\mu_{k}}\left[\sum_{n=1}^{N_{j,m}}\gamma^{n-1}r_{k,n}\right],$$

where γ is a constant that reflects the degree of discount applied to the long-term reward.

## 5. Solution

In this section, we first introduce the training process, which is based on the DDPG algorithm. Then, we describe how the performance of the trained model is tested in the testing stage.

### 5.1. Training Stage

Since the DDPG algorithm is capable of solving problems with the continuous action space, we employ the DDPG algorithm to obtain the optimal policy.

The DDPG algorithm combines the deterministic policy gradient (DPG) approach with the actor–critic framework; it is a modification of the DQN algorithm and can solve problems with the continuous action space. The DDPG algorithm is composed of four neural networks: an actor network, a target actor network, a critic network, and a target network. Here, the actor network and target actor network are employed to update the policy $\mu_{\theta^{k}}$, thus obtaining the optimal policy. The critic network and target critic network are employed to evaluate the policy.

The flow of the training stage is summarized in Algorithm 1. Note that $\theta^{k}$ and $\theta^{k^{\prime }}$ denote the parameters of the actor network and target actor network, respectively. $\zeta^{k}$ and $\zeta^{k^{\prime }}$ denote the parameters of the critic network and target critic network, respectively. $\Delta_{n}$ is the noise parameter in slot *n*.
**Algorithm 1:** Model training stage based on the DDPG algorithm
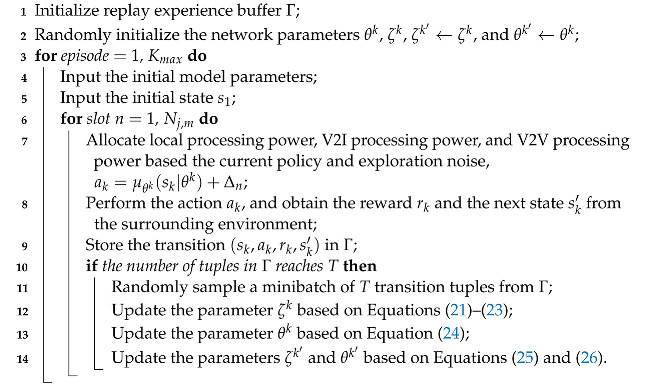


In the training stage, we randomly initialize $\theta^{k}$ and $\zeta^{k}$, while $\theta^{k^{\prime }}$ and $\zeta^{k^{\prime }}$ have the same initial values as $\theta^{k}$ and $\zeta^{k}$, respectively. Then, we define an experience buffer Γ with sufficient space to store the transitions for each slot (lines 1–2).

Without loss of generality, let us consider that model training starts for SVU *k*. In the first episode, the position of SVU *k* is first reset to within the range of the BS antennae. $d_{k,j}\left(1\right)$ is initialized as $-\frac{D}{2}$, and $B_{k}\left(1\right)$ is initialized as half of the buffer capacity. Then, $g_{k}^{s}\left(0\right)$ is randomly initialized and $g_{k}^{G}\left(0\right)$ is calculated by using Equation (9). Meanwhile, the SINR $\gamma_{k,I}\left(0\right)$ of SVU *k* for V2I communication is calculated by using Equation (8). Thus, the state of SVU *k* in slot 1 is obtained, i.e., $s_{k,1}=\left[B_{k}\left(1\right),\gamma_{k,I}\left(0\right),d_{k,j}\left(1\right)\right]$ (lines 3–5).

Subsequently, given the initial input $s_{k,1}$ to the actor network in slot 1, the corresponding policy $\mu_{\theta^{k}}\left(s_{k,1}|\theta^{k}\right)$ is obtained. The noise $\Delta_{1}$ is randomly initialized and then SVU *k* performs an action $a_{k,1}$ based on the current policy and the noise, $\mu_{\theta^{k}}\left(s_{k,1}|\theta^{k}\right)+\Delta_{1}$. With the performed action, the local processing power $p_{k,L}\left(1\right)$, the V2I processing power $p_{k,I}\left(1\right)$, and the V2V processing power $p_{k,V}\left(1\right)$ are determined. Then, SVU *k* obtains a reward $r_{k}\left(1\right)$ in accordance with Equation (19). The SINR $\gamma_{k,I}\left(0\right)$ for V2I communication is obtained in accordance with Equations (3)–(9), and $B_{k}\left(2\right)$ is obtained from Equations (12)–(16), where $d_{k,L}\left(1\right)$, $d_{k,I}\left(1\right)$, and $d_{k,V}\left(1\right)$ are obtained from Equation (13), (15) and (16), respectively. Additionally, $d_{k,j}\left(2\right)$ is obtained from Equation (2). Thus, the state of SVU *k* in slot 2 is obtained, i.e., $s_{k,2}=\left[B_{k}\left(2\right),\gamma_{k,I}\left(1\right),d_{k,j}\left(2\right)\right]$. Then, the tuple $\left(s_{k,1},a_{k,1},r_{k,1},s_{k,2}\right)$ is stored in the experience buffer Γ. If the number of tuples in Γ does not exceed *T*, SVU *k* proceeds to the next slot and repeats the above process (lines 6–10).

Once the number of tuples reaches *T*, the parameters $\theta^{k}$, $\zeta^{k}$, $\theta^{k^{\prime }}$, and $\zeta^{k^{\prime }}$ are updated toward maximizing $J(\mu_{\theta^{k}})$. The parameter $\theta^{k}$ is updated in accordance with the gradient of the policy, i.e., the gradient in the direction of $J(\mu_{\theta^{k}})$, which is denoted by $\nabla_{\theta^{k}}J\left(\mu_{\theta^{k}}\right)$. Let $Q^{\zeta^{k}}\left(s_{k,n},a_{k,n}\right)$ be the action value function of SVU *k*, which is the output of the critic network. According to [46], the task of solving for $\nabla_{\theta^{k}}J\left(\mu_{\theta^{k}}\right)$ can be replaced by solving for the gradient of $Q^{\zeta^{k}}\left(s_{k,n},a_{k,n}\right)$.

**Figure 2:**
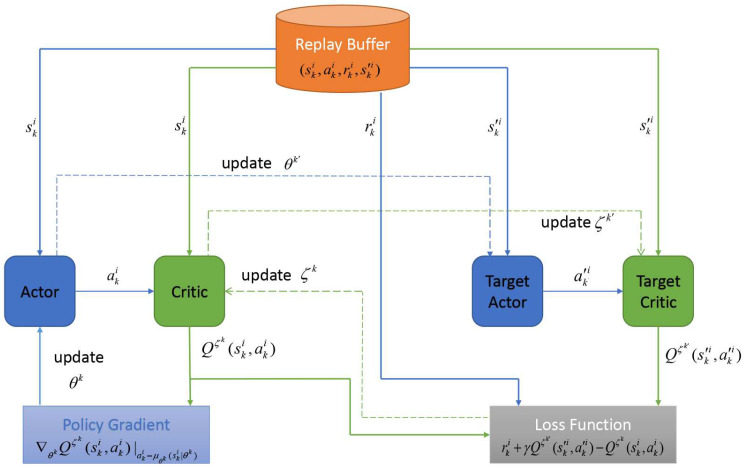
Flow chart of the DDPG algorithm.

Now, we further describe how the parameters are updated, as follows. Figure 2 illustrates the parameter update process of the DDPG algorithm. First, SVU *k* randomly selects *T* tuples from the experience buffer to form a minibatch. For convenience, we use $r_{k}$, $s_{k}$, $a_{k}$, $s_{k}^{\prime }$, and $a_{k}^{\prime }$ to denote $r_{k,n}$, $s_{k,n}$, $a_{k,n}$, $s_{k,n+1}$, and $a_{k,n+1}$, respectively. Then, let $\left(s_{k}^{i},a_{k}^{i},r_{k}^{i},{s^{\prime }}_{k}^{i}\right)$ denote the *i*th tuple in the minibatch. For the *i*th tuple, SVU *k* inputs ${s^{\prime }}_{k}^{i}$ into the target actor network, which outputs ${a^{\prime }}_{k}^{i}$ based on ${s^{\prime }}_{k}^{i}$. Then SVU *k* inputs ${s^{\prime }}_{k}^{i}$ and ${a^{\prime }}_{k}^{i}$ into the target critic network, which outputs the action value function $Q^{\zeta^{k^{\prime }}}\left({s^{\prime }}_{k}^{i},{a^{\prime }}_{k}^{i}\right)$. Thus, the target value of tuple *i* can be calculated as (line 11)

(21) $$y_{k}^{i}=r_{k}^{i}+\gamma Q^{\zeta^{k^{\prime }}}\left({s^{\prime }}_{k}^{i},{a^{\prime }}_{k}^{i}\right){|}_{{a^{\prime }}_{k}^{i}=\mu_{\theta^{k^{\prime }}}\left({s^{\prime }}_{k}^{i}|\theta^{k^{\prime }}\right)}.$$

then, $s_{k}^{i}$ and $a_{k}^{i}$ are used as inputs to the critic network, which outputs the action value function $Q^{\zeta^{k}}\left(s_{k}^{i},a_{k}^{i}\right)$. Thus, the loss function for tuple *i* can be expressed as

(22) $$L_{i}={\left[y_{k}^{i}-Q^{\zeta^{k}}\left(s_{k}^{i},a_{k}^{i}\right)\right]}^{2}.$$

accordingly, the loss function for all tuples can be expressed as

(23) $$L\left(\zeta^{k}\right)=\frac{1}{T}\sum_{i=1}^{T}L_{i}.$$

Then, SVU *k* updates $\zeta^{k}$ based on Equations (21)–(23) [47] (line 12). Afterward, $\nabla_{\theta^{k}}J\left(\mu_{\theta^{k}}\right)$ can be obtained from $Q^{\zeta^{k}}\left(s_{k}^{i},a_{k}^{\mu }\right)$, which is the output of the critic network. We have

(24) $$\begin{array}{cc} \; & \nabla_{\theta^{k}}J\left(\mu_{\theta^{k}}\right)\\ \; & \approx \frac{1}{T}\sum_{i=1}^{T}\nabla_{\theta^{k}}Q^{\zeta^{k}}\left(s_{k}^{i},a_{k}^{\mu }\right){|}_{a_{k}^{\mu }=\mu_{\theta^{k}}\left(s_{k}^{i}|\theta^{k}\right)}\\ \; & =\frac{1}{T}\sum_{i=1}^{I}\nabla_{a_{k}^{\mu }}Q^{\zeta^{k}}\left(s_{k}^{i},a_{k}^{\mu }\right){|}_{a_{k}^{\mu }=\mu_{\theta^{k}}\left(s_{k}^{i}|\theta^{k}\right)}\\ & \;\cdot \nabla_{\theta^{k}}\mu_{\theta^{k}}\left(s_{k}^{i}|\theta^{k}\right). \end{array}$$

note that the chain rule is utilized here, since $a_{k}^{\mu }=\mu_{\theta^{k}}\left(s_{k}^{i}|\theta^{k}\right)$ is an input for $Q^{\zeta^{k}}\left(s_{k}^{i},a_{k}^{\mu }\right)$.

Similarly, SVU *k* updates the parameter of the actor network in accordance with Equation (24) [47] (line 13).

In slot $N_{j,m}$, SVU *k* updates $\zeta^{k^{\prime }}$ and $\theta^{k^{\prime }}$, i.e., (line 14)

(25) $$\zeta^{k^{\prime }}\leftarrow \tau \zeta^{k}+\left(1-\tau \right)\zeta^{k^{\prime }},$$

(26) $$\theta^{k^{\prime }}\leftarrow \tau \theta^{k}+\left(1-\tau \right)\theta^{k^{\prime }},$$

where τ≪ 1 is a constant.

Finally, SVU *k* proceeds to the next slot and uses $s_{k}^{\prime }$ as the input to the actor network. This current episode continues until slot $N_{j,m}$ is reached. When the number of episodes reaches $K_{max}$, training of the system model is complete.

### 5.2. Testing Stage

In the testing stage, we test the performance of the trained system model. Algorithm 2 shows the flow of the testing process.
**Algorithm 2:**Testing stage for the trained model
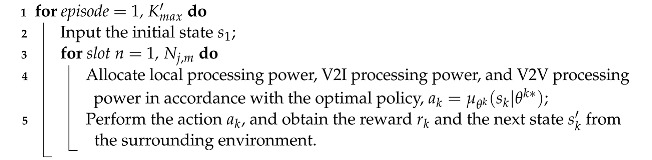


## 6. Simulation Results and Analysis

In this section, we demonstrate through simulation experiments that our proposed DDPG algorithm can obtain the optimal policy. The simulation experiments are divided into a training stage and a testing stage. The simulation tool is Python 3.7.

The key parameters of the experiments are listed in Table 2. The learning rates for the actor network and critic network are $10^{-3}$ and $10^{-4}$, respectively [47]. The size of the experience replay buffer is |Γ|. Task arrival follows a Poisson process, where the mean arrival rate is $\lambda_{k}$. SVU *k* is driving in lane 2, and its V2I communication will be interfered with three other vehicles when $d_{k,j}\left(n\right)=0$.

### 6.1. Training Stage

**Figure 3:**
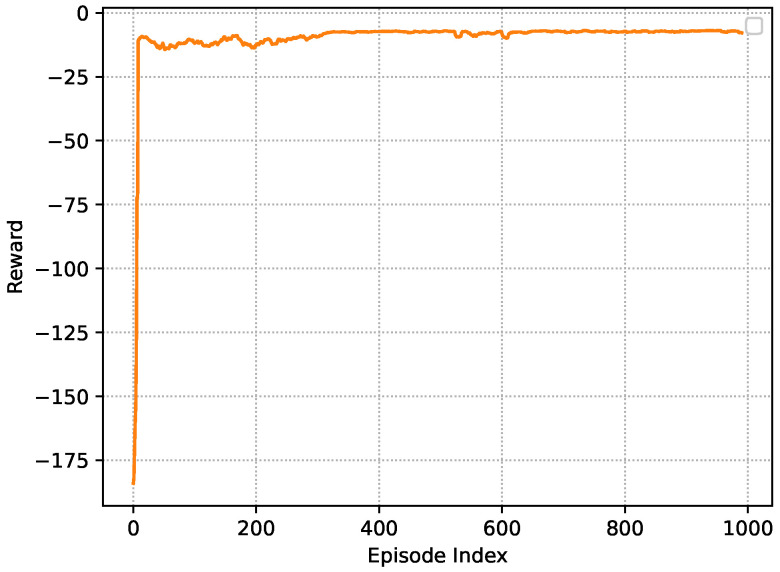
Learning curve in the training stage.

Figure 3 shows the learning curve in the training stage, where the reward is the average value in each episode. One can see that the average reward rises quickly from episode 0 to episode 12. Then, the curve declines from episode 12 to episode 400. This reflects that SVU *k* is adjusting its policy toward the optimal reward. From episode 400 to episode 1000, the rewards become stable with little jitter. The reason for the existence of jitter is the presence of exploration noise, which prevents SVU *k* from falling into a local optimum.

### 6.2. Testing Stage

**Figure 4:**
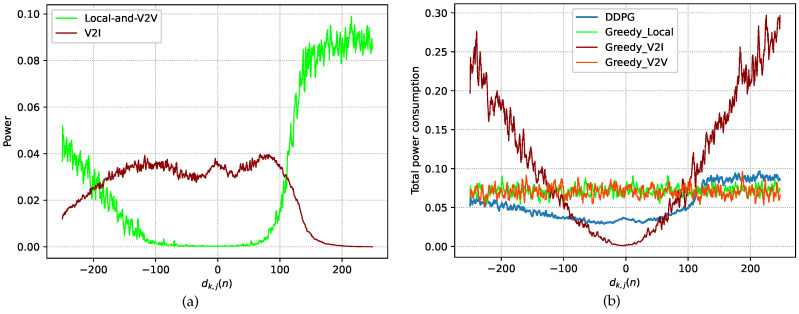
Power. (**a**) DDPG power allocation. (**b**) Total power consumption.

**Figure 5:**
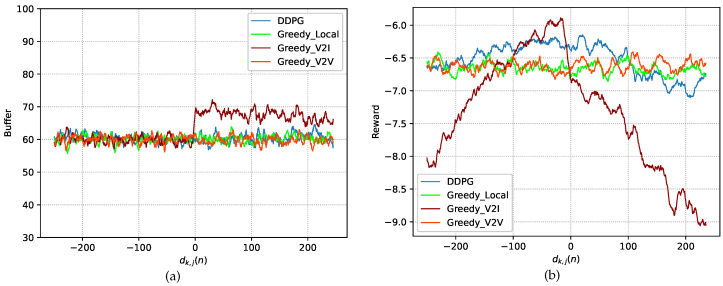
Performance. (**a**) Buffer capacity. (**b**) Reward.

We present performance tests performed on the trained model in the testing stage. Figure 4 and Figure 5 show the test performance under the DDPG algorithm and three other greedy (GD) policies in terms of the power consumption, buffer capacity, and reward, where the performance is recorded as the average value over 10 episodes. In the following, we introduce the three policies.

- GD-Local policy: SVU *k* first maximally allocates the local processing in each slot. The remaining tasks are equally allocated to V2I processing and V2V processing.
- GD-V2I policy: SVU *k* first maximally allocates the V2I processing power in each slot. The remaining tasks are equally allocated to local processing and V2V processing.
- GD-V2V policy: SVU *k* first maximally allocates the V2V processing power in each slot. The remaining tasks are equally allocated to local processing and V2I processing.

Figure 4a shows the power allocation under the DDPG algorithm at different distances, and Figure 4b compares the power consumption under all four policies at different distances. Figure 4a shows the local-and-V2V processing power and the V2I processing power at different distances under the DDPG algorithm. When $d_{k,j}\left(n\right)<0$, the V2I processing power increases, and the local-and-V2V processing power decreases significantly. This is because the path loss decreases; thus, the channel conditions for V2I communication improve as SVU *k* approaches the BS. Therefore, as SVU *k* approaches the BS, SVU *k* allocates more power to V2I processing. When SVU *k* moves away from the BS, SVU *k* allocates more power to local-and-V2V processing. When $d_{k,j}\left(n\right)>0$, local-and-V2V processing power increases rapidly, while the V2I processing power decreases. This is because when $d_{k,j}\left(n\right)=0$, the other three vehicles impose interference on SVU *k*, causing the V2I channel conditions to deteriorate. As the V2I channel conditions become worse, more power needs to be allocated to local-and-V2V processing. From Figure 4b, it can be seen that the power consumption under the GD-V2I policy gradually decreases when $d_{k,j}\left(n\right)<0$ and rapidly increases when $d_{k,j}\left(n\right)>0$, which clearly matches the changing V2I channel conditions.

Figure 5a,b compares the buffer capacity and reward under the four policies at different distances. From Figure 5a, it can be seen that the buffer capacity increases when $d_{k,j}\left(n\right)=0$ under the GD-V2I policy. This is because the V2I communication is interfered with by the other three vehicles when $d_{k,j}\left(n\right)=0$. In contrast, the buffer capacity under the DDPG algorithm remains stable. This means that SVU *k* is able to process tasks in a timely manner even though the channel conditions are degraded. As shown in Figure 5a, the reward under the DDPG algorithm is better than the rewards under the other three policies most of the time.

**Figure 6:**
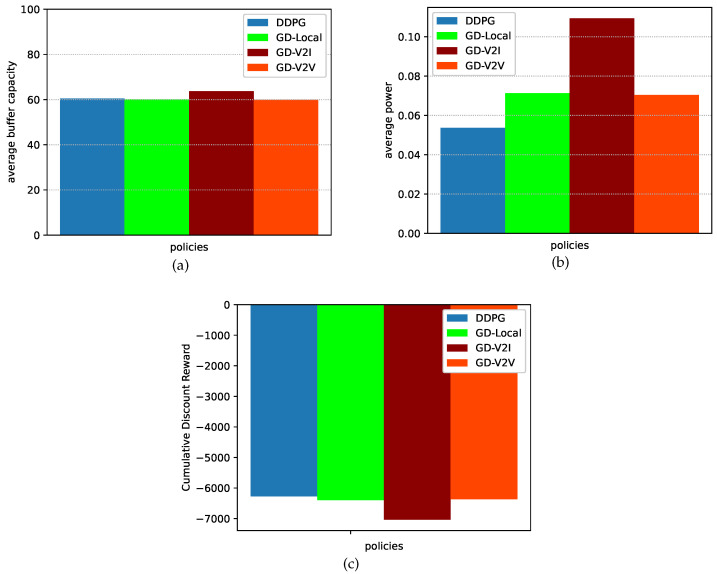
Performance. (**a**) Average buffer capacity. (**b**) Average power consumption. (**c**) Cumulative discount reward.

Figure 6a compares the average buffer capacity under the four policies. There is no significant difference in buffer capacity among the four policies. Nevertheless, the GD-V2I policy has the largest buffer capacity because of variation of V2I channel conditions. Meanwhile, as seen from Figure 6b, the average power consumption under the DDPG algorithm is significantly superior to the other three policies. Compared to that under the GD-Local policy, the average power consumption under the DDPG algorithm is reduced by 24.4%. Similarly, the average power reduction under the DDPG algorithm is 51.3% compared to the GD-V2I policy and 23.1% compared to the GD-V2V policy.

Figure 6c shows the cumulative discount reward under the four policies. We can see that the cumulative discount reward under the DDPG algorithm is better than those under the other three policies. This is because of the adaptability of the DDPG algorithm, which allows the model to rapidly adjust the power allocation.

**Figure 7:**
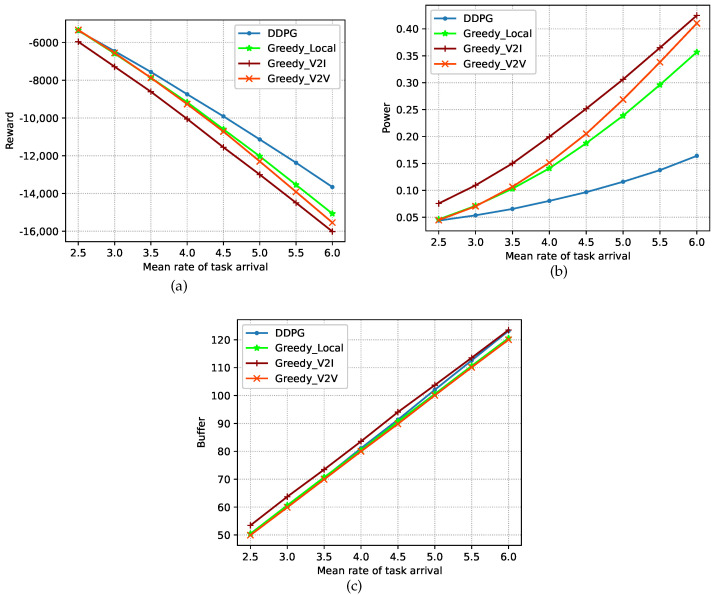
Performance at different task-arrival rates. (**a**) Cumulative discount reward. (**b**) Power consumption. (**c**) Buffer capacity.

Figure 7a,c shows the cumulative discount reward, power consumption, and buffer capacity under the four policies at different task-arrival rates. As the task-arrival rate increases, the cumulative discount reward decreases, the power consumption increases, and the buffer capacity becomes larger for all four policies. It can also be seen that although the DDPG algorithm is superior to the other three policies in terms of the power consumption and cumulative discount reward, its buffer capacity is slightly higher than those under the GD-Local and GD-V2V policies. This is because the DDPG algorithm aims to obtain the maximum cumulative discount reward. Due to its focus on power consumption, and the fact that its buffer capacity performance was not given more attention, the result is slightly worse.

Table 3 compares the performance in terms of average power consumption, average buffer capacity and cumulative discount reward under the four policies, where A, B, C, and D stand for different performance levels in a descending order.

## 7. Conclusions

In this paper, we have proposed a decentralized DRL-based VEC power allocation model that considers not only the coexistence of D2D-based V2V communication and MIMO-NOMA-based V2I communication, but also the mobility of SVUs, the randomness of task arrival, and the channels’ interference in MIMO-NOMA. Extensive simulations demonstrate that the average power consumption and reward under the DDPG algorithm is superior to those of other policies. Meanwhile, since the proposed DDPG algorithm focuses on power consumption, it may incur a compromise for buffer capacity. For future work, we will consider the coexistence of many-to-many D2D and MIMO-NOMA technology in VEC systems.

## Figures and Tables

**Table 1:** Notations used in this article.

Notation	Description	Notation	Description
$a_{k,n}$	Action of SVU *k* in slot *n*.	$a_{k}\left(n\right)$	Number of task bits of SVU *k* arriving in slot *n*.
$a_{k}$	Abbreviation for $a_{k,n}$.	$a_{k}^{i}$	Action of the *i*th tuple.
$a_{k}^{\prime }$	Abbreviation for $a_{k,n+1}$.	${a^{\prime }}_{k}^{i}$	Next action of the *i*th tuple.
$\beta_{0}$	Channel power gain at the reference distance.	$B_{k}\left(n\right)$	Buffer capacity of SVU *k* in slot *n*.
*D*	Diameter of the BS’s range.	e(n)	Error vector.
$d_{k,L}\left(n\right)$	Number of task bits processed locally by SVU *k* in slot *n*.	$d_{k,I}\left(n\right)$	Number of task bits processed for SVU *k* performing V2I communication in slot *n*.
$d_{k,j}\left(n\right)$	Distance between SVU *k* and the BS along the *x*-axis in slot *n*.	$d_{k,V}\left(n\right)$	Number of task bits processed for SVU *k* performing V2V communication in slot *n*.
$f_{i}$	An exponentially distributed random variable with unit mean.	$f_{k}\left(n\right)$	CPU frequency of SVU *k* in slot *n*.
$g_{k}^{s}\left(n\right)$	Small-scale fading channel gain of SVU *k* in slot *n*.	$g_{k,I}\left(n\right)$	Channel vector of SVU *k* for V2V communication in slot *n*.
$g_{k}^{p}\left(n\right)$	Large-scale fading coefficient for V2I communication at slot *n*.	$g_{r}$	Channel power gain at 1 m.
G(n)	MIMO-NOMA channel martix.	$h_{k,V}$	Channel gain of SVU *k* for V2V communication in slot *n*.
$J(\mu_{k})$	Objective function.	$K_{max}$	Maximum number of episodes in the training stage.
$L(\zeta^{k})$	Loss function.	*L*	Number of CPU cycles for processing one bit.
$N_{s}\left(n\right)$	Noise received by the BS.	$N_{j,m}$	Last slot in lane *j*.
$N_{\max,j}$	Maximum number of SVUs in lane *j*.	$N_{a}$	Number of antennae.
$P_{k,j}\left(n\right)$	Location of SVU *k* in slot *n*.	$P_{B}$	Position of the BS antenna.
$p_{k,I}\left(n\right)$	Processing power of SVU *k* for V2I communication in slot *n*.	$p_{k,L}\left(n\right)$	Local processing power of SVU *k* in slot *n*.
$p_{k,V}\left(n\right)$	Processing power of SVU *k* for V2V communication in slot *n*.	$P_{max,V}$	Maximum V2I processing power.
$P_{max,I}$	Maximum V2I processing power.	$P_{max,L}$	Maximum local processing power.
$Q^{\mu_{\theta^{k}}}\left(s_{k,n},a_{k,n}\right)$	Action value function of SVU *k*.	$Q^{\zeta^{k}}\left(s_{k,n},a_{k,n}\right)$	Action value function output from the critic network.
$Q^{\zeta^{k^{\prime }}}\left(s_{k,n},a_{k,n}\right)$	Action value function output from the target critic network.	$r_{k,n}$	Reward of SVU *k* in slot *n*.
$r_{k}$	Abbreviation for $r_{k,n}$.	$r_{k}^{i}$	Reward of the *i*th tuple.
Γ	Experience buffer.	$s_{k,n}$	State space of SVU *k* in slot *n*.
$s_{k}$	Abbreviation for $s_{k,n}$.	$s_{k}^{i}$	State of the *i*th tuple.
$s_{k}^{\prime }$	Abbreviation for $s_{k,n+1}$.	${s^{\prime }}_{k}^{i}$	Next state of the *i*th tuple.
*T*	Maximum number of tuples in a minibatch.	$v_{k}$	Velocity of SVU *k*.
$v_{j}$	Velocity of lane *j*.	$w_{1}$	Distance from lane 1 to the BS.
$W_{d}$	Bandwidth.	$w_{k,j}$	Distance between SVU *k* driving in lane *j* and antennas along the *y*-axis.
$w_{d}$	Lane width.	$y_{k}^{i}$	Target value of the *i*th tuple.
y(n)	Signal received by the BS.	$\alpha_{k}^{C}$	Learning rate of the critic network.
$\alpha_{k}^{A}$	Learning rate of the actor network.	$\alpha_{h}$	Path loss exponent for V2V communication.
$\rho_{k}$	Normalized channel correlation coefficient of SVU *k*.	$\lambda_{k}$	Mean rate of task arrival for SVU *k*.
$\zeta^{k}$	Parameter of the critic network.	$\zeta^{k^{\prime }}$	Parameter of the target critic network.
κ	Effective converted capacitance of SVU *k*.	$R_{k}$	Distance between SVU *k* and the corresponding VEC vehicle.
$\theta^{k}$	Parameter of the actor network.	$\theta^{k^{\prime }}$	Parameter of the target actor network.
γ	Discount factor.	$\Delta_{n}$	Exploration noise in slot *n*.
$\mu_{\theta^{k}}$	Policy of SVU *k* approximated by the actor network.	$\mu_{k}^{*}$	Optimal policy of SVU *k*.
$\sigma_{R}^{2}$	Variance of the Gaussian noise in communication.	τ	Update degree parameter for the target networks.
$\tau_{0}$	Slot duration.	$\omega_{1},\omega_{2}$	Reward weight factors.
$\gamma_{k,I}\left(n\right)$	SINR of SVU *k* for V2I communication in slot *n*.	η	Path loss exponent.
$\gamma_{k,V}\left(n\right)$	SINR of SVU *k* for V2V communication in slot *n*.		

**Table 2:** Key parameters in simulations.

**Parameters of the System Model**			
**Parameter**	**Value**	**Parameter**	**Value**
$\sigma_{R}^{2}$	$10^{-9}$ W	$h_{r}$	−30 dB
$\beta_{0}$	−30 dB	$W_{d}$	1 MHZ
$\tau_{0}$	20 ms	κ	$10^{-28}$
$v_{1}$	20 m/s	$v_{2}$	25 m/s
$v_{3}$	30 m/s	$w_{d}$	10 m
*L*	500 cycles/bit	$\lambda_{k}$	3 Mbps
*H*	10 m	$N_{a}$	4
*D*	500 m	$P_{max,V}$	1 W
$P_{max,I}$	1 W	$P_{max,L}$	1 W
$R_{k}$	65 m	$w_{1}$	10 m
$\alpha_{h}$	2		
**Parameters of the Training Process**			
**Parameter**	**Value**	**Parameter**	**Value**
$\alpha_{k}^{C}$	0.001	$\alpha_{k}^{A}$	0.0001
$\omega_{1}$	0.9	$\omega_{2}$	0.1
γ	0.99	τ	0.001
$K_{max}$	1000	*T*	64
$K_{max}^{\prime }$	10	|Γ|	$2.5\times 10^{5}$

**Table 3:** Performance comparison under the four policies.

Policies	Average Power Consumption	Average Buffer Capacity	Cumulative Discount Reward
DDPG	*A*	*B*	*A*
GD-Local	*B*	*B*	*B*
GD-V2I	*D*	*C*	*D*
GD-V2V	*B*	*B*	*B*

## References

[1] Raza S., Wang S., Ahmed M., Anwar M.R., Mirza M.A., Khan W.U. (2022). Task Offloading and Resource Allocation for IoV Using 5G NR-V2X Communication. IEEE Internet Things J..

[2] Wu Q., Wan Z., Fan Q., Fan P., Wang J. (2022). Velocity-Adaptive Access Scheme for MEC-Assisted Platooning Networks: Access Fairness Via Data Freshness. IEEE Internet Things J..

[3] Wu Q., Xia S., Fan Q., Li Z. (2019). Performance Analysis of IEEE 802.11p for Continuous Backoff Freezing in IoV. Electronics.

[4] Wu Q., Zheng J. (2015). Performance Modeling and Analysis of IEEE 802.11 DCF Based Fair Channel Access for Vehicle-to-Roadside Communication in a Non-Saturated State. Wirel. Netw..

[5] Sabireen H., Neelanarayanan V. (2021). A Review on Fog Computing: Architecture, Fog with IoT, Algorithms and Research Challenges. ICT Express.

[6] Zhang X., Zhang J., Liu Z., Cui Q., Tao X., Wang S. (2020). MDP-Based Task Offloading for Vehicular Edge Computing Under Certain and Uncertain Transition Probabilities. IEEE Trans. Veh. Technol..

[7] Zhang K., Mao Y., Leng S., He Y., Zhang Y. (2017). Mobile-Edge Computing for Vehicular Networks: A Promising Network Paradigm with Predictive Off-Loading. IEEE Veh. Technol. Mag..

[8] Wu Q., Zhao Y., Fan Q., Fan P., Wang J., Zhang C. (2022). Mobility-Aware Cooperative Caching in Vehicular Edge Computing Based on Asynchronous Federated and Deep Reinforcement Learning. IEEE J. Sel. Top. Signal Process..

[9] Hou X., Li Y., Chen M., Wu D., Jin D., Chen S. (2016). Vehicular Fog Computing: A Viewpoint of Vehicles as the Infrastructures. IEEE Trans. Veh. Technol..

[10] Hou X., Ren Z., Wang J., Cheng W., Ren Y., Chen K.-C., Zhang H. (2020). Reliable Computation Offloading for Edge-Computing-Enabled Software-Defined IoV. IEEE Internet Things J..

[11] Zhu H., Wu Q., Wu X.-J., Fan Q., Fan P., Wang J. (2022). Decentralized Power Allocation for MIMO-NOMA Vehicular Edge Computing Based on Deep Reinforcement Learning. IEEE Internet Things J..

[12] Asadi A., Wang Q., Mancuso V. (2014). A Survey on Device-to-Device Communication in Cellular Networks. IEEE Commun. Surv. Tut..

[13] Ren Y., Liu F., Liu Z., Wang C., Ji Y. (2015). Power Control in D2D-Based Vehicular Communication Networks. IEEE Trans. Veh. Technol..

[14] Sun W., Yuan D., Ström E.G., Brännström F. (2016). Cluster-Based Radio Resource Management for D2D-Supported Safety-Critical V2X Communications. IEEE Trans. Wirel. Commun..

[15] Sun W., Ström E.G., Brännström F., Sou K.C., Sui Y. (2016). Radio Resource Management for D2D-Based V2V Communication. IEEE Trans. Veh. Technol..

[16] Nguyen K.K., Duong T.Q., Vien N.A., Le-Khac N.-A., Nguyen L.D. (2019). Distributed Deep Deterministic Policy Gradient for Power Allocation Control in D2D-Based V2V Communications. IEEE Access.

[17] Wu Q., Shi S., Wan Z., Fan Q., Fan P., Zhang C. (2022). Towards V2I Age-aware Fairness Access: A DQN Based Intelligent Vehicular Node Training and Test Method. Chin. J. Electron..

[18] Wang H., Ke H., Liu G., Sun W. (2020). Computation Migration and Resource Allocation in Heterogeneous Vehicular Networks: A Deep Reinforcement Learning Approach. IEEE Access.

[19] Dong P., Ning Z., Ma R., Wang X., Hu X., Hu B. (2020). NOMA-based energy-efficient task scheduling in vehicular edge computing networks: A self-imitation learning-based approach. China Commun..

[20] Wang Q., Fan P., Letaief K.B. (2012). On the Joint V2I and V2V Schedule for Cooperative VANET with Network Codeding. IEEE Trans. Veh. Technol..

[21] He Y., Zhao N., Yin H. (2018). Integrated Networking, Caching, and Computing for Connected Vehicles: A Deep Reinforcement Learning Approach. IEEE Trans. Veh. Technol..

[22] Luo Q., Li C., Luan T.H., Shi W. (2020). Collaborative Data Scheduling for Vehicular Edge Computing via Deep Reinforcement Learning. IEEE Internet Things J..

[23] Liu Y., Yu H., Xie S., Zhang Y. (2019). Deep Reinforcement Learning for Offloading and Resource Allocation in Vehicle Edge Computing and Networks. IEEE Trans. Veh. Technol..

[24] Tan L.T., Hu R.Q. (2018). Mobility-Aware Edge Caching and Computing in Vehicle Networks: A Deep Reinforcement Learning. IEEE Trans. Veh. Technol..

[25] Zhu Z., Wan S., Fan P., Letaief K.B. (2022). Federated Multiagent Actor–Critic Learning for Age Sensitive Mobile-Edge Computing. IEEE Internet Things J..

[26] Wu Q., Zhao Y., Fan Q. (2022). Time-Dependent Performance Modeling for Platooning Communications at Intersection. IEEE Internet Things J..

[27] Hai T., Zhou J., Padmavathy T.V., Md A.Q., Jawawi D.N.A., Aksoy M. (2022). Design and Validation of Lifetime Extension Low Latency MAC Protocol (LELLMAC) for Wireless Sensor Networks Using a Hybrid Algorithm. Sustainability.

[28] Wu Q., Liu H., Zhang C., Fan Q., Li Z., Wang K. (2019). Trajectory protection schemes based on a gravity mobility model in iot. Electronics.

[29] Wang K., Yu F., Wang L., Li J., Zhao N., Guan Q., Li B., Wu Q. (2019). Interference alignment with adaptive power allocation in full-duplex-enabled small cell networks. IEEE Trans. Veh. Technol..

[30] Fan J., Yin S., Wu Q., Gao F. Study on refined deployment of wireless mesh sensor network. Proceedings of the 2010 6th International Conference on Wireless Communications Networking and Mobile Computing (WiCOM).

[31] Ye H., Li G.Y., Juang B.-H.F. (2019). Deep Reinforcement Learning Based Resource Allocation for V2V Communications. IEEE Trans. Veh. Technol..

[32] Xu Y.-H., Yang C.-C., Hua M., Zhou W. (2020). Deep Deterministic Policy Gradient (DDPG)-Based Resource Allocation Scheme for NOMA Vehicular Communications. IEEE Access.

[33] Ding C., Wang J.-B., Zhang H., Lin M., Wang J. (2021). Joint MU-MIMO Precoding and Resource Allocation for Mobile-Edge Computing. IEEE Trans. Wirel. Commun..

[34] Liu Y., Cai Y., Liu A., Zhao M., Hanzo L. (2022). Latency Minimization for mmWave D2D Mobile Edge Computing Systems: Joint Task Allocation and Hybrid Beamforming Design. IEEE Trans. Veh. Technol..

[35] Li Y., Xu G., Yang K., Ge J., Liu P., Jin Z. (2020). Energy Efficient Relay Selection and Resource Allocation in D2D-Enabled Mobile Edge Computing. IEEE Trans. Veh. Technol..

[36] Zhang H., Wang Z., Liu K. (2020). V2X offloading and resource allocation in SDN-assisted MEC-based vehicular networks. China Commun..

[37] Bai X., Chen S., Shi Y., Liang C., Lv X. Collaborative Task Processing in Vehicular Edge Computing Networks. Proceedings of the 2021 4th International Conference on Hot Information-Centric Networking (HotICN).

[38] Ning Z., Zhang K., Wang X., Obaidat M.S., Guo L., Hu X., Hu B., Guo Y., Sadoun B., Kwok R.Y.K. (2021). Joint Computing and Caching in 5G-Envisioned Internet of Vehicles: A Deep Reinforcement Learning-Based Traffic Control System. IEEE Trans. Intell. Transp..

[39] Ren T., Yu X., Chen X., Guo S., Xue-Song Q. Vehicular Network Edge Intelligent Management: A Deep Deterministic Policy Gradient Approach for Service Offloading Decision. Proceedings of the 2020 International Wireless Communications and Mobile Computing (IWCMC).

[40] Jang Y., Na J., Jeong S., Kang J. Energy-Efficient Task Offloading for Vehicular Edge Computing: Joint Optimization of Offloading and Bit Allocation. Proceedings of the 2020 IEEE 91st Vehicular Technology Conference (VTC2020-Spring).

[41] Zhan W., Luo C., Wang J., Wang C., Min G., Duan H., Zhu Q. (2020). Deep-Reinforcement-Learning-Based Offloading Scheduling for Vehicular Edge Computing. IEEE Internet Things J..

[42] Ngo H.Q., Larsson E.G., Marzetta T.L. (2013). Energy and Spectral Efficiency of Very Large Multiuser MIMO Systems. IEEE Trans. Commun..

[43] Abramowitz M., Stegun I.A. (1988). Handbook of Mathematical Functions: With Formulas, Graphs, and Mathematical Tables. Am. J. Phys..

[44] Kwak J., Kim Y., Lee J., Chong S. (2015). DREAM: Dynamic Resource and Task Allocation for Energy Minimization in Mobile Cloud Systems. IEEE J. Sel. Area. Comm..

[45] King C. Fundamentals of wireless communications. Proceedings of the 2014 IEEE-IAS/PCA Cement Industry Technical Conference.

[46] Silver D., Lever G., Heess N., Degris T., Riedmiller M. Deterministic Policy Gradient Algorithms. Proceedings of the 2014 International Conference on Machine Learning(ICML).

[47] Lillicrap T.P., Hunt J.J., Pritzel A., Heess N., Erez T., Tassa Y., Silver D., Wierstra D. (2015). Continuous control with deep reinforcement learning. arXiv.

